# Accuracy of a commercial multiplex PCR for the diagnosis of bacterial vaginosis

**DOI:** 10.1099/jmm.0.000792

**Published:** 2018-07-09

**Authors:** Charlotte van der Veer, Robin van Houdt, Alje van Dam, Henry de Vries, Sylvia Bruisten

**Affiliations:** ^1^​ Department of Infectious Diseases, Public Health Service Amsterdam, Amsterdam, the Netherlands; ^2^​ Department of Medical Microbiology and Infection Prevention, VU University Medical Center, Amsterdam, the Netherlands; ^3^​ Department of Medical Microbiology, OLVG General Hospital, Amsterdam, the Netherlands; ^4^​ Department of Dermatology, Academic Medical Center, University of Amsterdam, Amsterdam, the Netherlands; ^5^​ Amsterdam Infection and Immunity Institute, Academic Medical Center, University of Amsterdam, Amsterdam, the Netherlands

**Keywords:** bacterial vaginosis, molecular diagnostics, multiplex PCR, vaginal microbiota

## Abstract

**Purpose:**

Bacterial vaginosis (BV) is a common clinical condition characterized by odorous vaginal discharge, vaginal itching and/or burning. BV can occur when vaginal lactobacilli are depleted and replaced by diverse anaerobic bacteria. We evaluated a commercial multiplex PCR (ATRiDA) for the diagnosis of BV.

**Methods:**

Cervicovaginal samples were included from women reporting urogenital symptoms and from women notified for sexually transmitted infections (STI) – who were not (necessarily) symptomatic. Clinical BV diagnoses were obtained from electronic patient files. The ATRiDA test measures the loads of *Gardnerella vaginalis*, *Atopobium vaginae* and *Lactobacillus* species in relation to overall bacterial load. The ATRiDA test outcome was compared to the clinical BV diagnosis and to vaginal microbiota composition, determined by 16SrRNA gene sequencing.

**Results:**

We included samples from 185 women reporting urogenital symptoms, of whom 81 had BV and 93 women who were notified for an STI, of whom 16 had BV. Overall, compared to the clinical BV diagnosis, the ATRiDA test demonstrated high sensitivity (96.9 %) and moderate specificity (70.2 %). The negative predictive value was high (>97.3). The positive predictive value differed by study group and was highest in women reporting urogenital symptoms (78.2 %). Sequencing showed that 54 % of women who had an ATRiDA BV-positive test outcome, but who were not clinically diagnosed with BV, had diverse anaerobic vaginal microbiota (asymptomatic vaginal dysbiosis).

**Conclusion:**

The ATRiDA test is a sensitive method for the detection of BV but, given the high occurrence of asymptomatic vaginal dysbiosis, a positive test outcome should be interpreted together with clinical symptoms.

## Introduction

Bacterial vaginosis (BV) is a common, non-inflammatory clinical condition characterized by malodorous vaginal discharge, vaginal itching and/or a burning sensation [1]. During episodes of BV, the vaginal environment has lower abundance of lactobacilli and increased abundance of diverse anaerobic bacteria with or without *Lactobacillus iners*; however, a reduction in lactobacilli can occur asymptomatically and a vaginal dysbiotic state without the above-mentioned symptoms is not considered BV.

Several techniques have been developed for the diagnosis of BV. Since the 1950s a positive culture for *Gardnerella vaginalis* (then known as *Haemophilus vaginalis*) was considered indicative for what was then known as non-specific vaginitis [2]. In the 1980s, the term BV was coined and the Amsel criteria were introduced where a BV was confirmed when at least three of the following criteria were met: (1) thin, white–greyish homogenous vaginal discharge; (2) an elevated pH (≥4.5); (3) amine (fishy) odour after addition of 10 % potassium hydroxide (KOH) to a vaginal wet mount smear; and (4) presence of ‘clue’ cells: epithelial cells coated with bacteria [3]. In the 1990s a standardized scoring system was introduced, the Nugent score, where vaginal smears were gram stained and scored for the ratio of different bacterial morphologies [4]. In the 2000s, molecular tools were developed that targeted and semi-quantified *Lactobacillus* spp. and BV-associated bacteria [5–8]. Measuring the Amsel criteria and performing a Nugent score are relatively inexpensive, but both techniques are laborious and require experienced personnel. Molecular tools may therefore soon gain preference – especially in (Westernized) clinical settings where they can be afforded and implemented.

Here we evaluated a commercially developed and in-house validated multiplex PCR (ATRiDA) for the diagnosis of BV. We assessed the tool for its accuracy (sensitivity, specificity and negative and positive predictive values) in cervicovaginal samples from women who had attended the STI outpatient clinic in Amsterdam either because they had reported urogenital symptoms or because they had been notified for an STI by a sex partner, but were not (necessarily) symptomatic. We made a distinction between these two groups to evaluate the test’s performance in two groups of women that differed with respect to reporting urogenital symptoms.

## Methods

### Study populations and tests performed

All data used in this study were collected as part of routine management and anonymized before analysis. Clients were notified that remainders of their samples might be used for scientific research. If clients objected, data and samples were discarded. Thus, by Dutch law, no further ethical clearance was needed in this study. We collected cervicovaginal samples from female clients attending the STI outpatient clinic in Amsterdam, the Netherlands, who had self-reported any urogenital symptoms (March–July 2015) or who had been notified by a sex partner for *Chlamydia trachomatis* infection (September 2013–December 2014). All clients were asked about their medical and sexual history and underwent an extensive physical examination (including speculoscopy) [9]. Cervicovaginal swabs were collected for STI testing (nucleic acid amplification tests for *Chlamydia trachomatis*, *Neisseria gonorrhoeae* and *Trichomonas vaginalis* (APTIMA, Hologic, Marlborough, USA)). Direct microscopy of vaginal wet mounts and Gram stains were assessed for other causes of vaginitis, such as candidiasis and BV, by looking for: yeast cells, leukocytes, ‘clue’ cells and an amine (fishy) odour after addition of 10 % potassium hydroxide (KOH). Medical history, clinical findings (such as grey/white homogenous vaginal discharge) and microscopy results all contributed to the clinical diagnosis of BV. In this study we used the BV outcome as recorded in the electronic patient files, as clinical BV diagnosis to which the ATRiDA test was referenced.

### Sample collection and DNA extraction

Cervicovaginal swabs, collected for STI testing, were placed in transport medium (APTIMA, Hologic, Marlborough, USA) immediately after collection and processed according to the manufacturer’s protocol. DNA was extracted from 200 µl of these processed samples by isopropanol precipitation and the pellet was dissolved in 50 µl 10 mM Tris/HCL. This DNA was subsequently used for the ATRiDA test and targeted 16s rRNA gene sequencing.

### Multiplex PCR procedure for BV diagnosis

The ATRiDA test (ATRiDA B.V., Amersfoort, the Netherlands) targets the bacterial species *G. vaginalis*, *Atopobium vaginae*, *Lactobacillus* spp. and total bacteria. We performed the multiplex PCR according to the manufacturer’s protocol, but used a lower total volume for the PCR reaction: 15 µl instead of 25 µl, which was validated in samples with known BV status (data not shown). Volume reduction made the test more affordable in our routine setting. Each PCR reaction contained 6 µl FRT mix 1 and 2.7 µl FRT mix 2 with 10 % Taq polymerase, 3 µl molecular-grade water and 3 µl sample DNA. Each PCR run included a BV-positive and -negative control and two calibrator samples to quantify the relative bacterial load of each target within a sample. All PCRs were performed in a RotorGene machine (Qiagen, Hilden, Germany). A preformatted Excel sheet calculated bacterial ratios based on three coefficients: RC1=log (*Lactobacillus* spp.) − log (*A. vaginae+G. vaginalis*), RC2=log (bacteria) − log (*Lactobacillus* spp.) and RC3=log (bacteria) − log (*G. vaginalis+A. vaginae*). BV PCR outcomes were generated as follows: (1) if *Lactobacillus* spp. were predominant (RC1>1), the outcome was defined as BV negative; (2) if *A. vaginae* and *G. vaginalis* were present in large amounts (RC1 <0.5), the outcome was defined as BV positive; (3) if the RC was between 0.5 and 1.0, the outcome was defined as intermediate; and (4) if *Lactobacillus* spp. were *not* predominant and the cumulative concentration of *A. vaginae* and *G. vaginalis* was substantially lower than the total concentration of bacteria, the outcome was flora alteration, unspecified (RC2>1 and RC3>2).

### Vaginal microbiota composition, 16 s rRNA targeted sequencing

The vaginal microbiota composition was analysed by targeted sequencing of the 16S rRNA gene (V3–V4 region), as described previously [10]. In short, extracted DNA was amplified using the dual indexed universal primers 319F and 806R, pooled and sequenced on the illumina MiSeq platform (San Diego, USA). Only high-quality sequences (>99 % base call accuracy; Trimmomatic [11]) were retained and aligned using PandaSeq [12]. Aligned sequences were mapped to their corresponding bar codes using the demultiplex tool in QIIME (version 1.9) [13]. Operational taxonomic units (OTUs) were selected using the usearch tool in QIIME and aligned to a vaginal reference package developed by Srinivasan *et al.* [1,4] using PPLACER [15]. Relative abundances for each taxonomy per sample were calculated and a dendrogram based on the maximum distance in matrix dissimilarity between the relative abundances per sample was created using the ggplot2 package in R, version 3.2.1.

### ATRiDA BV multiplex PCR diagnostic accuracy

Sensitivity, specificity and positive and negative predictive values (PPV, NPV) were calculated for the ATRiDA test relative to the clinical BV diagnosis. In the contingency analyses, ATRiDA unspecified test results were excluded and the ATRiDA intermediate test results were grouped together with the BV negative results as this reflects clinical practice; intermediate results have the same clinical consequence as BV negative results. We also performed separate contingency analyses that used (combinations) of clinical parameters for BV reported in the electronic patient files, rather than the reported BV outcome, as reference methods.

### Statistical analysis

Chi-squared and Fisher's exact tests were used to compare demographic or clinical characteristics between women with and without BV. All tests were performed in SPSS Statistics software version 21 (IBM, New York, USA).

## Results

### Characteristics of the study populations

We included samples from 185 women reporting urogenital symptoms, of whom 81 received a clinical diagnosis for BV. The second group of women who were notified for an STI included 93 women, of whom 16 had BV. In both groups, women with and without BV were similar in regard to ethnic background, sexual orientation, number of sex partners and STI prevalence. In the symptomatic group, women without BV had candidiasis more often than women with BV (14.6 vs 4.8 %; *P*=0.031). Only 33.3 % of the STI-notified women reported urogenital symptoms, and this was significantly associated with having BV (*P*<0.001). See Table 1.

**Table 1.** Characteristics of women reporting urogenital symptoms or notified by a sex partner for *Chlamydia trachomatis* and attending the STI outpatient clinic in Amsterdam, the Netherlands, stratified by BV status based on the STI clinic BV diagnosis*

	Women reporting urogenital symptoms			Women notified by a sex partner for *Chlamydia trachomatis* infection		
	BV negative*	BV positive*	*P*-value†	BV negative*	BV positive*	*P*-value†
	*N*=104 (%)	*N*=81 (%)		*N*=77 (%)	*N*=16 (%)	
Age in years; median (IQR)	24.7 (21–27)	24.7 (21–28)	0.805	24.1 (19.7–27.2)	20.9 (18.9–22.4)	**0.028**
Ethnicity			0.214			0.924
European	78 (75.0)	54 (66.6)		52 (67.5)	11 (68.8)	
Non-European	26 (25.0)	27 (33.3)		25 (32.5)	5 (31.2)	
No. of sex partners in past 6 months median (IQR)	4 (2–4)	3 (1–3)	0.169	2 (1–3)	2 (1–2)	0.078
Heterosexual	97 (93.3)	78 (96.3)	0.517	77 (100.0)	16 (100.0)	1.00
Self-reported urogenital symptoms‡	104 (100)	81 (100)	1.00	18 (23.4)	13 (81.2)	**<0.001**
*Candida*	16 (15.4)	3 (3.7)	**0.009**	7 (9.1)	0 (0.0)	0.600
*Trichomonas vaginalis*	0 (0.0)	0 (0.0)	–	1 (1.3)	0 (0.0)	1.000
*Chlamydia trachomatis*	8 (7.7)	8 (9.9)	0.793	41 (53.2)	11 (68.8)	0.256
*Neisseria gonorrhoea*	1 (1.0)	1 (1.2)	1.000	2 (2.6)	2 (12.5)	0.076

*BV diagnosis outcome as reported in the electronic patient files.

†Chi-squared or Fisher’s exact (when expected count <5) test for categorical data and Mann–Whitney U test for continuous data.

‡Urogenital self-reported symptoms such as: pain, odorous discharge, itching, burning sensation, rash and purulence.

### Vaginal microbiota composition by study population and BV outcome

**Fig. 1.**
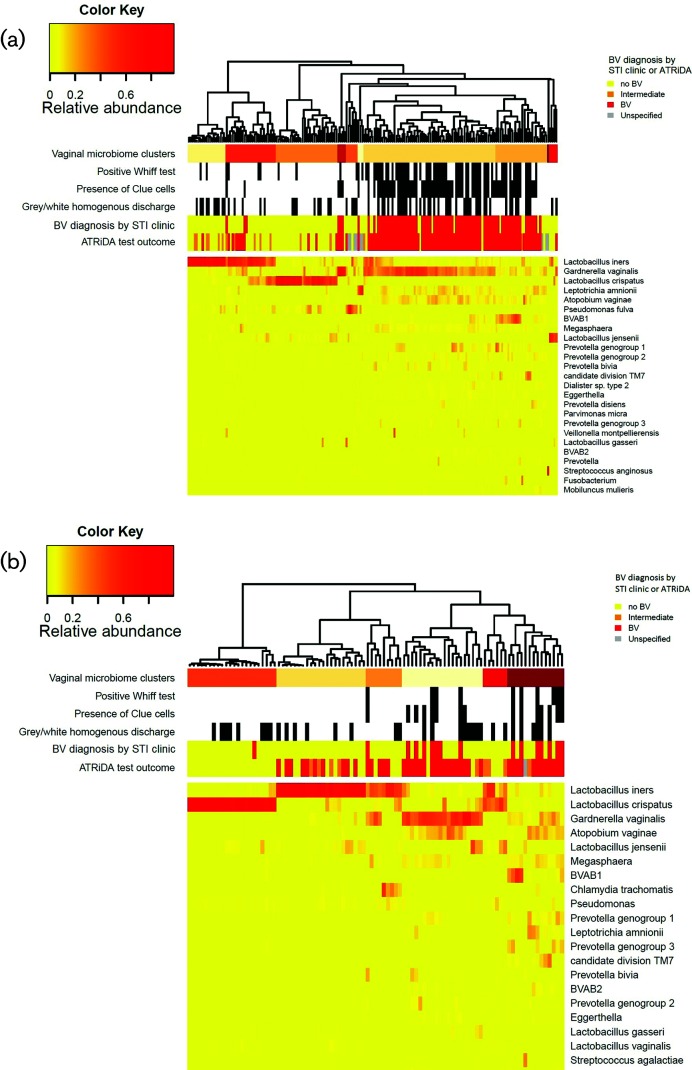
Heatmap depicting microbial species (y-axis) per sample (x-axis) of a) women reporting urogenital symptoms and b) women notified for an STI. Microbial relative abundance is illustrated by the colour key. Sidebars above the heatmap depict vaginal microbiome clusters (top bar), BV clinical parameters and clinical BV diagnosis as recorded in the electronic patient file (middle bars) and ATRiDA test outcome (bottom bar).

Fig. 1(a, b) displays the vaginal microbial composition as assessed by 16s rRNA gene sequencing of each sample by the clinical BV diagnosis as reported in the electronic patient files and the ATRiDA test outcome. Of note, over half of women who had an ATRiDA BV-positive test outcome, but who were not clinically diagnosed with BV, had diverse anaerobic vaginal microbiota (symptomatic group: *n*=10/22; 45.5 %; STI-notified group: *n*=18/29; 62.1 %), followed by *L. iners*-dominated microbiota (symptomatic group: *n*=8/45; 17.8 %; STI-notified group: *n*=11/26; 42.3 %).

### ATRiDA BV multiplex PCR diagnostic accuracy

We found an overall sensitivity and specificity for the ATRiDA test of respectively 96.9 and 70.2 %, relative to the clinical BV diagnosis (Table 2). The ATRiDA test’s performance was higher in the symptomatic group (sensitivity: 97.5 %; NPV: 97.3 %; specificity: 76.8 %; PPV: 77.5 %) than in the STI-notified group (sensitivity: 93.8 %; NPV: 97.9 %; specificity: 61.8 %; PPV: 34.1 %). Unspecified ATRiDA test results (symptomatic group: *n*=9; 4.8 %, STI-notified group: *n*=1; 0.7 %) were excluded from the analyses. When performing the contingency analyses using different (combinations) of clinical parameters for BV extracted from the electronic patient files as reference methods, the ATRiDA test’s sensitivity and NPV increased (>98.0 %) but the specificity and PPV decreased (respectively, >57.8 % and >20.5 %). See Tables S1–S3 (available in the online version of this article).

**Table 2.** ATRiDA test outcome accuracy as referenced to the STI clinic BV diagnosis in women with urogenital complaints or notified by a sex partner for *Chlamydia trachomatis* attending the STI outpatient clinic in Amsterdam, the Netherlands

	Women reporting urogenital symptoms	Women notified by a sex partner for *Chlamydia trachomatis* infection	Overall
	*N*=185, of whom 81 had a clinical diagnosis of BV*	*N*=93, of whom 16 had a clinical diagnosis of BV*	*N*=279, of whom 97 had a clinical diagnosis of BV*
Sensitivity† (%)	97.5 (*n*=79/81)	93.8 (*n*=15/16)	96.7 (*n*=94/97)
Specificity† (%)	76.8 (*n*=73/95)	61.8 (*n*=47/76)	70.2 (*n*=120/171)
NPV† (%)	97.3 (*n*=73/75)	97.9 (*n*=47/48)	–
PPV† (%)	78.2 (*n*=79/101)	34.1 (*n*=15/44)	–

*BV diagnostic outcome as reported in the electronic patient files.

†ATRiDA-intermediate results were grouped together with the BV-negative results and ATRiDA-unspecified results were excluded from the contingency analyses.

## Discussion

We evaluated the performance of a commercial multiplex BV PCR (ATRiDA) test in cervicovaginal samples from two groups of women that differed with respect to reporting urogenital symptoms. The first group of women all reported urogenital symptoms and therefore attended the STI clinic, whereas the second group had attended the STI clinic only after an STI partner notification and not (necessarily) because they were symptomatic. Given the overall high sensitivity (>96.9 %) and negative predictive value (>97.5 %) of the ATRiDA test, a negative test outcome is likely to be reliable. The relatively low specificity and PPV, and especially the difference in PPV when considering the reason for clinic visit (symptomatic group: 78.2 % vs STI notified group: 34.1 %) indicates that a positive ATRiDA test should always be interpreted together with clinical symptoms for BV.

The exact aetiology of BV remains unknown and this complicates the development of molecular tools for BV. Several anaerobic bacteria that associate with BV have been identified, including *G. vaginalis* and *A. vaginae*, but also *Megaspheara*, *Prevotella* and bacterial vaginosis-associated bacteria (BVAB) 1, 2 and 3 [5], though none of these bacteria are specific for BV and have also been found in women without BV [16]. Kusters *et al.* and Hilbert *et al.* both developed an in-house qPCR that targeted *Megaspheara* in addition to *G. vaginalis* and *A. vaginae* [6, 7]. Both studies reported a much higher specificity (>95 %), but a slightly lower sensitivity (>92 %), than reported here. This shows that the specificity of a molecular test can indeed be increased by including an extra BV-associated target, though at a slight cost to sensitivity. The test developed by Kusters *et al.* [7] also included a *Lactobacillus* species index, comparing *L. iners* and *L. crispatus* ratios, which aided the BV definitive diagnosis when only one BV-associated bacterial species was detected. While *L. crispatus* has consistently been found to be protective against BV and STI, by for example inhibiting epithelial adhesion of other organisms [17, 18], *L. iners* is often found in high numbers in women with BV and STIs [10, 19, 20]. *L. iners* is therefore considered less beneficiary or, at least, less resilient against vaginal disturbances that could lead to BV [21]. The ATRiDA test did not differentiate between the various *Lactobacillus* species, although we saw that *L. crispatus*-dominated vaginal samples were almost always BV-negative, whereas almost one-third of *L. iners-*dominated vaginal samples tested positive for BV (Fig. 1). *L. iners*-dominated samples sometimes also contained some *G. vaginalis*, which could explain the BV-positive ATRiDA test outcomes for those samples.

Cross-sectional studies of asymptomatic women have shown that those of European descent were more likely than women of non-European descent to have *L. crispatus-*dominated vaginal microbiota, whereas the latter were more likely to have vaginal microbiota dominated by *L. iners* [22–24]. Moreover, vaginal dysbiosis (defined here as vaginal microbiota not dominated by *Lactobacillus* spp.) was most common among women of African descent [22–24]. Twenty-five percent of the women included in our study were of non-European descent, which could have influenced the specificity of the ATRiDA test. Rumyantseva *et al.* [8] also evaluated the ATRiDA test in clinical samples from Swedish women diagnosed by Amsel criteria and they reported a sensitivity of 100 % and a specificity of 91 %, though they did not mention the ethnic background of their study participants [8]. Of note, our study differed from that of Rumyantseva *et al.* [8] in that we referenced the ATRiDA test to the BV outcome as reported in the electronic patient file and this outcome did not strictly adhere to the Amsel criteria, primarily because the vaginal pH was not measured. Thomason *et al.* [25], however, have shown that vaginal pH had a low PPV (52.6 %; i.e. vaginal pH may increase for reasons other than BV) and of the four Amsel criteria, the presence of ‘clue’ cells and a positive ‘whiff’ test were statistically the most significant predictors of BV (NPV: 92.1 % and PPV: 98.8 %). To support our findings, we performed separate contingency analyses that used (combinations of) the presence of ‘clue’ cells and/or a positive ‘whiff’ test as reference methods, rather than the reported BV outcome. In these separate analyses we observed a similar general performance – high sensitivity (>98.0 %) but low specificity (>57.8 %). See Tables S1–S3.

We attempted to differentiate between symptomatic and asymptomatic women based on self-reported urogenital complaints. However, a self-reported symptom is a limitation *per se* since women may experience symptoms differently. Moreover, our study sampled from one STI clinic population only and this may not be representative of other populations who are, for example, at lower risk for STI. Vaginal dysbiosis is associated with (recent) sexual activity and especially with new sexual partners [22, 26], which could mean that we underestimated the ATRiDA test’s specificity in our study. The specificity could have been further underestimated by our use of cervicovaginal samples, while BV is a condition that affects the vaginal epithelium. Moreover, fluctuations in the vaginal microbiota composition and/or stability of the microbial composition were shown to occur during or around the time of menstruation, but the microbial composition often restores itself after menstruation [27]. We had no information on menses, so it is possible that some samples that tested positive for BV with the ATRiDA test were taken around the time of menstruation and may have reflected a naturally transient vaginal dysbiotic state. Vaginal dysbiosis is a risk factor for acquiring STI [10, 17, 19] and for having adverse pregnancy outcomes such as preterm labour [28]. Pregnancy status was unknown in our STI clinic population. Further prospective investigation is needed to assess whether the ATRiDA test could be used as a screening tool in pregnant women or in women at risk for STI. The test is suitable for laboratories equipped for molecular testing. We have reduced the overall cost by minimizing the amount of reagents needed per test; however, molecular testing in general is expensive and in low-income settings cheaper options such as Nugent scoring and Amsel criteria might be more feasible for the diagnosis of BV.

In conclusion, this commercially available BV multiplex PCR (ATRiDA) is a sensitive method with a good negative predictive value in the detection of BV. Because of the relatively low specificity, explained by the high occurrence of asymptomatic vaginal dysbiosis, a positive test outcome should always be interpreted in combination with a clinical case definition for BV. Furthermore, it is recommended that a molecular diagnostic test for BV should be performed only in women with a suspicion of BV based on their clinical presentation, and these tests should not be performed as a screening tool in other asymptomatic women.

## Supplementary Data

Supplementary File 1
