# Supplementary material for: Accuracy of a commercial multiplex PCR for the diagnosis of bacterial vaginosis
Source: J Med Microbiol. 2018 Jul 9;67(9):1265–70. doi: 10.1099/jmm.0.000792 (PMC6230723; doi:10.1099/jmm.0.000792)
Supplement: Supplementary File 1 [file jmm-67-1265-s001.pdf]

Supplementary Tables

Supplementary Table 1. ATRiDA test outcome accuracy as referenced to the clinical parameters: positive for ‘clue cells and ‘whiff’ test, as recorded in the electronic patients files in women with urogenital complaints or notified by a sex partner for Chlamydia trachomatis attending the STI outpatient clinic in Amsterdam, the Netherlands.

	**Women with urogenital symptoms (n=185, of whom 57 had a clinical diagnosis for BV[1])**	**Women notified by a sex partner for *Chlamydia trachomatis* infection (n=93, of whom 9 had a clinical diagnosis for BV[1])**
Sensitivity[2]	98.2 (n=56/57)	100 (n=9/9)
Specificity[2]	62.2 (n=74/119)	57.8 (n=48/83)
NPV[2]	98.7 (n=74/75)	100 (n=48/48)
PPV[2]	55.4 (n=56/101)	20.5 (n=9/44)

[1] BV diagnosis was considered positive when ‘clue’ cells were detected and a positive ‘whiff’ test.

[2] Intermediate results were grouped together with the BV negative results and unspecified results were excluded from the contingency analyses.

Supplementary Table 2. ATRiDA test outcome accuracy as referenced to the clinical parameters: positive for 'clue cells, as recorded in the electronic patients files in women with urogenital complaints or notified by a sex partner for Chlamydia trachomatis attending the STI outpatient clinic in Amsterdam, the Netherlands.

	**Women with urogenital symptoms (n=186, of whom 77 had a clinical diagnosis for BV[1])**	**Women notified by a sex partner for *Chlamydia trachomatis* infection (n=93, of whom 15 had a clinical diagnosis for BV[1])**
Sensitivity[2]	98.7 (n=76/77)	100 (n=15/15)
Specificity[2]	74.7 (n=74/99)	62.3 (n=48/77)
NPV[2]	98.7 (n=74/75)	100 (n=48/48)
PPV[2]	75.2 (n=76/101)	34.1 (n=15/44)

[1] BV diagnosis was considered positive when 'clue' cells were detected.

[2] Intermediate results were grouped together with the BV negative results and unspecified results were excluded from the contingency analyses.

Supplementary Table 3. ATRiDA test outcome accuracy as referenced to the clinical parameters: positive for 'clue cells and 'whiff' test or white/grey homogenous discharge, as recorded in the electronic patients files in women with urogenital complaints or notified by a sex partner for Chlamydia trachomatis attending the STI outpatient clinic in Amsterdam, the Netherlands.

	**Women with urogenital symptoms (n=186, of whom 64 had a clinical diagnosis for BV[1])**	**Women notified by a sex partner for *Chlamydia trachomatis* infection (n=93, of whom 9 had a clinical diagnosis for BV[1])**
Sensitivity[2]	98.4 (n=63/64)	100 (n=9/9)
Specificity[2]	61.2 (n=74/121)	57.8 (n=48/83)
NPV[2]	98.7 (n=74/75)	100 (n=48/48)
PPV[2]	62.3 (n=63/101)	20.5 (n=9/44)

[1] BV diagnosis was considered positive when 'clue' cells were detected and a positive 'whiff' test and/or white/grey homogenous discharge was detected.

[2] Intermediate results were grouped together with the BV negative results and unspecified results were excluded from the contingency analyses.
